# Beyond Psychometrics: The Difference between Difficult Problem Solving and Complex Problem Solving

**DOI:** 10.3389/fpsyg.2017.01739

**Published:** 2017-10-10

**Authors:** Jens F. Beckmann, Damian P. Birney, Natassia Goode

**Affiliations:** ^1^School of Education, Durham University, Durham, United Kingdom; ^2^School of Psychology, University of Sydney, Sydney, NSW, Australia; ^3^Centre for Human Factors and Sociotechnical Systems, University of the Sunshine Coast, Sunshine Coast, QLD, Australia

**Keywords:** complex problem solving, semantic effect, complexity vs. difficulty, systematicity, person–task–situation

## Abstract

In this paper we argue that a synthesis of findings across the various sub-areas of research in complex problem solving and consequently progress in theory building is hampered by an insufficient differentiation of complexity and difficulty. In the proposed framework of person, task, and situation (PTS), complexity is conceptualized as a quality that is determined by the cognitive demands that the characteristics of the task and the situation impose. Difficulty represents the quantifiable level of a person’s success in dealing with such demands. We use the well-documented “semantic effect” as an exemplar for testing some of the conceptual assumptions derived from the PTS framework. We demonstrate how a differentiation between complexity and difficulty can help take beyond a potentially too narrowly defined psychometric perspective and subsequently gain a better understanding of the cognitive mechanisms behind this effect. In an empirical study a total of 240 university students were randomly allocated to one of four conditions. The four conditions resulted from contrasting the semanticity level of the variable labels used in the CPS system (high vs. low) and two instruction conditions for how to explore the CPS system’s causal structure (starting with the assumption that all relationships between variables existed vs. starting with the assumption that none of the relationships existed). The variation in the instruction aimed at inducing knowledge acquisition processes of either (1) systematic elimination of presumptions, or (2) systematic compilation of a mental representation of the causal structure underpinning the system. Results indicate that (a) it is more complex to adopt a “blank slate” perspective under high semanticity as it requires processes of inhibiting prior assumptions, and (b) it seems more difficult to employ a systematic heuristic when testing against presumptions. In combination, situational characteristics, such as the semanticity of variable labels, have the potential to trigger qualitatively different tasks. Failing to differentiate between ‘task’ and ‘situation’ as independent sources of complexity and treating complexity and difficulty synonymously threaten the validity of performance scores obtained in CPS research.

## Introduction

Complex problem solving (CPS) is an umbrella term for a diverse range of approaches to research, learning and assessment. A common denominator of all these approaches is the use of a computerized simulation of some abstract or contextualized system. This is where the main commonality ends. However, when considering the various different ways problem solvers can interact with these simulations and the wide variety of different purposes of their use, it becomes apparent that the term CPS has many different meanings. One meaning refers to a *research paradigm* that aims to study “complex” cognition in the context of information processing, decision-making, causal reasoning, or learning (Beckmann, 1994; Beckmann and Guthke, 1995; Frensch and Funke, 1995; Guthke et al., 1995). In other domains (e.g., Greiff et al., 2015), CPS has been considered as an *ability-related construct* (or set of constructs). One example is the ability to deal with uncertainty (e.g., Osman, 2010) with its conceptual – yet not always empirically aligned – links to reasoning and (fluid) intelligence (Funke and Frensch, 2007; Stadler et al., 2015). CPS has also started to establish its use in relation to an assessment approach, be it in smaller-scale studies in relation to personnel decisions (Wood et al., 2009) or in relation to larger-scale educational attainment assessment exercises such as PISA (OECD, 2013; Funke et al., 2017). Within an assessment context, CPS is often discussed as a *skill or competency* (rather than ability). On the one hand, the shared use of the term CPS in these contexts tends to belie the conceptual, and concomitantly, methodological diversity in this field of research; on the other hand, such diversity in meaning raises the suspicion of an insufficient conceptual foundation of CPS.

As a look beyond CPS and at scientific theory building paradigms generally reminds us, a lack of conceptual grounding tends to result in definitions for the respective target constructs that are predominantly operational (rather than conceptual). This is evident in the CPS literature, as CPS has often been used as a descriptor of the kind of *behavior* observable when individuals are confronted with a specific kind of challenge (i.e., CPS is what problem solvers do when dealing with complex problems). As a corollary of a preponderance of operational definitions, research and subsequent publications seem to be heavily focussing on psychometric characteristics of CPS simulations as measurement tools. In its extreme, such a situation might be perceived as delegating conceptual decisions to statistical procedures.

With this paper, we aim to go beyond the psychometrically driven approach to CPS and to contribute to a more theory-based positioning of it within a nomological network of cognition. Our argumentation leads to an empirical investigation that explicitly differentiates manipulations of complexity (the conceptual) from experiences of difficulty (the psychometric) and in so doing, demonstrate the importance of separating statistical and conceptual issues in the investigation of CPS.

## Complexity vs. Difficulty

One symptom of a predominantly psychometric view on CPS is the lack of a distinction between complexity and difficulty (Beckmann and Goode, 2017). Difficulty is a psychometric concept with rather limited *explanatory* value. In general terms, difficulty provides a *descriptive* account of some items being answered correctly by a smaller proportion of individuals than other items, thus creating the basis for them being labeled as more difficult. When interested in the reasons for their higher levels of difficulty, one is confronted with a tautological reference (common to classical-test-theory) to the lower proportion of correct responses these items tend to attract. Actual explanations as to why this might be the case, however, need to go beyond such circularity. An analysis of the cognitive behavior required to tackle the problem posed by an item, as well as a reflection of the circumstances under which the item is expected to be solved, feeds into the notion of complexity. In this regard, complexity reflects ex ante considerations of the cognitive demands imposed by the task and the circumstances under which the task is to be performed (i.e., predictions), which makes complexity a primarily *cognitive concept*. Difficulty is experiential, person-bound and by definition, statistical. It is a reflection of how well individuals (with their individual differences in ability, knowledge, skills, motivation, etc.) deal with complexity, which makes it a *psychometric concept*.

At a first and rather pragmatic glance, such distinction may seem pedantic. After all, so it could be claimed, the presumptions linking difficulty (statistics) with complexity (theory) are built on a wealth of replicable scientific evidence. Therefore, so one might argue, our criticism would be considered not only unfounded in practice, but even counterproductive to the pursuit of knowledge. However, it is very easy to demonstrate this is not the case, and that when considered through person–task–situation (PTS) interactions, the broader CPS paradigm proves to be particularly in need of such a distinction. In the following we present a framework that allows for a conceptual differentiation between complexity and difficulty in the context of CPS. We then empirically test core arguments derived from this framework.

We start by taking the perspective of CPS as a research paradigm that utilizes computerized scenarios as task stimuli. These computerized scenarios or microworlds can be conceptualized as systems (e.g., Funke, 1985, 1992). In their simplest form, such systems comprise two kinds of variables that are causally linked, input variables and output variables and the interconnectedness of these system variables can be algorithmically described through linear structural equations. Such systems are considered “dynamic” if output variables change both as an effect of problem solvers inputs and independently over time.

In the contexts of research, assessment and learning, problem solvers are usually asked to first explore the unknown causal structure of these systems. In general, such an *exploration phase* serves the purpose of knowledge acquisition. In a subsequent *control phase*, problem solvers are then asked to reach and maintain pre-defined goal states in the output variables. The objective here is the application or utilization of the knowledge acquired during the exploration phase. In the terminology of generic problem solving, the typical CPS constellation is where a particular set of operations has to be identified (i.e., knowledge acquired) that will bring the system from a given initial state to a set target one (i.e., system control).

## CPS in the Three Dimensional Space of Person, Task, and Situation (PTS)

As has been discussed previously (Beckmann, 2010; Birney et al., 2016; Beckmann and Goode, 2017), psychological research takes place in the three dimensional space of Person, Task, and Situation variables. The definition of task has two sub-facets, the *task qua task* and the *task as behavior requirement* (McGrath and Altman, 1966; Hackman, 1969; Wood, 1986). The *task qua task* facet refers to the physical characteristics of the stimuli the problem solver is confronted with. In the context of CPS, these are the characteristics of the CPS scenario which include, but are not limited to, the number of variables or the density of their interconnectedness. The *task as behavior requirement* refers to what the problem solver is instructed to do. In the context of CPS, this could be, for example, to either freely interact with the given system to uncover its causal structure, or to control this system, i.e., to reach and maintain a set of target states in the output variables. Both, task qua task and task as behavior requirements contribute interactively to the complexity of the CPS task. That is, being confronted with the same system (*task qua task*) but with different instructions – as communicated *task as behavior requirement* – results in different tasks with different levels of complexity. In short, different tasks require different sets of *expected*^1^ (cognitive) behavior and therefore contribute differently to complexity.

The definition of *situation* refers to the environment or the circumstances in which a given task is to be performed (“task environment” as described by Newell and Simon, 1972, p. 55). In the context of CPS this includes situational characteristics such as whether a causal diagram (i.e., a graphical representation of the causal structure) is available or not when being asked to control the system. Knowing or being able to anticipate the target states during the exploration phase (or not) would be another situational characteristic. As these and other variations in circumstantial characteristics are also expected to result in differing sets of cognitive behaviors (despite being confronted with the same system and the same instruction), the situation is conceptualized as another contributor to complexity.

So far we have identified that both the task (with its two facets *task qua task* and *task as behavior requirement*) and the situation contribute to complexity. The third category of variables is linked to *Person* and includes, inter alia, individual differences in reasoning ability, information processing capacity, motivation, working memory, experience, and knowledge. Observed performance is the resultant of the *difficulty* individuals’ experience in dealing with the complexity imposed by the task and the situation. In short, difficulty is the observable, subjective reflection of complexity.

Experimental research in psychology, irrespective of its focus, builds on observing variation in one component of this tripartite system of variables (i.e., Person, Task, and Situation) whilst the variation in the other two is either controlled for or, more or less systematically manipulated. The dominant experimental paradigms can be defined by their focus on one of these three components. For instance, in an *assessment* context, test takers are confronted with a standardized set of tasks under standardized instructions (e.g., to control a particular microworld) and in standardized situations (e.g., after a knowledge acquisition phase that resulted in a causal diagram, which is made available on the computer screen). Standardization ensures that all test takers are dealing with the same level of complexity (as it is defined by the system, the instructed task and the situation), so that observed variability in performance scores between (and occasionally within) individuals can be attributed to individual differences in conceptually relevant person characteristics (e.g., reasoning ability).

In comparison, in the context of *cognition research*, participants are confronted with systems (task qua task) or situations that differ systematically as part of experimental manipulations. Randomization in the allocation of participants to conditions aims at controlling for potential effects of individual differences. This allows for observed variability in average performances scores across conditions to be attributed to differences in complexity caused by the variation in task and/or situation characteristics.

In the context of *instructional design* research, as another example, the situational features of a (learning) task are systematically varied (e.g., availability and location of information – say, 0, 1, or more mouse-clicks away) whilst the task as behavior requirements (e.g., to acquire structural knowledge) and the task qua task (i.e., the system) are kept the same across learners. Observed performance differences are then interpreted as indications of how various *situational* variables (e.g., interface features) make a learning task more or less complex.

In sum, complexity and difficulty are different concepts. Failing to differentiate between the two is problematic in at least two ways. First, equating (observation-based and psychometrically derived) difficulty with complexity serves to perpetuate the circular argument of that what is difficult must be complex, and what makes something complex is its difficulty. Second, equating (task and situation analysis based) complexity with difficulty creates the dilemma of not being able to “explain” why the same level of complexity (as set by the task and the situation) results in different individuals experiencing varying levels of difficulty (as observed via differences in performance scores). The first problem creates the risk of a tautological trap that is often associated with operational definitions of constructs; the second problem seems to negate the role of the individual or person and therefore promotes a rather “un-psychological” perspective *per se*. For CPS to be taken beyond a predominantly psychometric approach, the differentiation between complexity and difficulty is a necessary precondition. Otherwise, by remaining too narrowly focussed at a psychometric level, CPS could just as appropriately be labeled Difficult Problem Solving (i.e., DPS) – a term which can be readily recognized as data driven and theoretically vacuous.

Projecting CPS-related research and its findings onto the tripartite system of Person, Task, and Situation as briefly outlined above provides a framework for the necessary differentiation between complexity and difficulty. In the following we use the semantic effect (Beckmann, 1994; Beckmann and Guthke, 1995; Beckmann and Goode, 2014), as an exemplar for how the PTS-based differentiation between complexity and difficulty can help take CPS beyond a psychometric perspective and subsequently gain a better understanding of the cognitive mechanisms behind this effect.

## Variable Labels as Situational Characteristic – A Source of Complexity or Difficulty?

Previous research has repeatedly shown that seemingly minor changes of situational characteristics such as using semantically laden labels for system variables in comparison to semantically neutral labels have profound effects on performance (Beckmann, 1994; Beckmann and Guthke, 1995; Beckmann and Goode, 2014). In these studies, problem solvers tend to acquire less knowledge and subsequently control the system rather poorly when the same system is presented as a ‘Cherry Tree’ with input variables labeled ‘Light,’ ‘Water,’ and ‘Temperature’ linked to output variables labeled ‘Cherries,’ ‘Leaves,’ and ‘Beetles’ in comparison to a ‘Machine’ with input variables labeled as control dials ‘A,’ ‘B,’ ‘C’ and output variables labeled display ‘X,’ ‘Y,’ ‘Z.’ This phenomenon was initially described as the ‘Semantic Effect’ (e.g., Beckmann, 1994).

Projecting the semantic effect onto the tripartite framework of Person, Task, and Situation (PTS) implies that whilst presenting problem solvers with the same system (i.e., keeping the *task qua task* constant) and instructing them to execute the same tasks (i.e., keeping *tasks as behavior requirements* constant) still creates systematic variability in performance (i.e., indicating differences in difficulty) when a *situational characteristic* (e.g., the semantic meaning of variable labels) is varied.

As previous research has suggested, problem solvers confronted with system labels high in semanticity tend to approach the task of exploring a complex, dynamic system with a set of presumptions regarding the interrelatedness of system variables, whilst problem solvers working with variable labels low in semanticity tend to start with a “blank slate” concerning the causal structure of the system (Beckmann and Goode, 2014). In the former situation, knowledge acquisition would require a process of systematically *eliminating* presumed, yet not existing relationships and therefore reducing the complexity of the internal representation of the system’s causal structure. In the latter situation, knowledge acquisition from a “blank slate” perspective would require a process of systematically *compiling* knowledge and therefore building up the complexity of the internal representation of the system’s causal structure. Predicting whether the cognitive processes involved in eliminating presumptions are *more complex* than those in relation to compiling knowledge would be challenging from a purely psychometric perspective.

Concomitantly, the observed performance differences in the context of the semantic effect are associated with differences in the *systematicity* of the exploration behavior (Beckmann and Goode, 2014). Systematicity in exploration behavior is reflected in a specific sequence of interventions. First, all inputs are left at zero. Any changes in the outputs can then be interpreted as autonomic changes (i.e., eigendynamics). Subsequent interventions should then focus on the effects of each input variable on any of the output variables in isolation, i.e., changing only one input at a time. Only such “Vary-One-or-None-At-a-Time” heuristic (VONAT, see Beckmann and Goode, 2014; p. 279; Beckmann and Goode, 2017) creates informative system state transitions that allow problem solvers to derive knowledge regarding the causal structure of the system. In contrast, changing more than one variable at a time or to miss the zero change intervention creates what de Jong and van Joolingen (1998, p. 185) describe as “inconclusive experiments,” which impedes successful knowledge acquisition.

## Aims and Hypotheses

We use the phenomenon of the semantic effect as an exemplary case for testing the conceptual assumption derived from the PTS framework that situational variables – in addition to task variables – present a potential source of complexity.

First, and based on findings from previous research (e.g., Beckmann and Goode, 2014) we expect problem solvers working with variable labels high in semanticity to be less systematic in their exploration behavior (*Systematicity Hypothesis*). We then address the question whether the inferior CPS performance observed under semantically rich conditions (i.e., the semantic effect) can be explained by (1) supposedly higher cognitive demands associated with a process of reducing the complexity of an internal representation of the causal structure of the explored system, or by (2) problem solvers “simply” not employing the appropriate heuristic (i.e., systematically testing against a priori assumptions). In the context of the PTS framework, results in accordance to (1) would recommend situational variables as contributors to *complexity*; results in accordance to (2) would suggest that situational variables contribute to the *difficulty* of dealing with a complex dynamic system.

The validity of a conceptual distinction between complexity and difficulty, which is based on the PTS framework, can be tested by observing the effect of explicitly instructing problem solvers to systematically explore the system by either starting with the presumption that all relationships exist (thus requiring to eliminate non-existing relationships and to reduce the complexity of the mental representation of the causal structure) or by starting with the presumption that no relationships exist (thus requiring to compile the set of relationships that exist and to build up the complexity of the mental representation of the causal structure). If eliminating presumptions to arrive at the correct model of the system is *more complex* (via imposing greater demands on cognitive behavior) than starting with a blank slate then performance scores should worsen (both knowledge and control performance). Should, however, performance scores improve, this would suggest that problem solvers fail to engage in cognitive behavior that they are in fact capable of (*Complexity–Difficulty Hypothesis*). In psychometric terminology, the latter outcome would suggest that semanticity has the potential of introducing “construct-irrelevant difficulty” (Messick, 1995), and therefore represents a threat to validity.

## Methods

### Participants

The sample comprised of 240 students from two Australian universities across a wide range of subjects, including engineering, business studies, science related subjects and medicine (60% female, mean age 22.7 years).

### Materials

**Figure A1:**
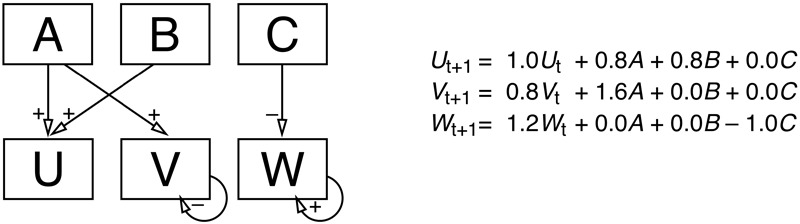
Diagram and set of equations for the causal structure of the CPS system used in this study.

To test both the Semanticity-Hypothesis and the Complexity–Difficulty Hypothesis four different versions of a CPS scenario with three input and three output variables were created (**Figure A1** shows the causal diagram and the underpinning equations that govern this system). These four versions were embedded in a 2 (semanticity: high vs. low) by 2 (instruction: compile vs. eliminate) between subject design. In the two high semanticity versions, variable labels related to a Cherry Tree were used (i.e., ‘HEAT,’ ‘LIGHT,’ and ‘WATER’ for the input variables and ‘CHERRIES,’ ‘LEAVES,’ and ‘BEETLES’ for the output variables). In the two low semanticity versions, variables low in semantic value were used (i.e., ‘INPUT A,’ ‘INPUT B,’ and ‘INPUT C’ and ‘OUTPUT U,’ ‘OUTPUT V,’ and ‘OUTPUT W,’ respectively), referring to a ‘BLACK BOX.’ For each of the two semanticity conditions two instruction conditions were created. In the compile conditions problem solvers were instructed to explore the causal structure of the given system by starting with ‘… the assumption that no relationship existed, and to systematically find out which of the possible links do, in fact, exist.’ In the eliminate conditions problem solvers were instructed to explore the causal structure of the system by starting with ‘… the assumption that all the relationships existed, and to systematically find out which of the possible links do, in fact, not exist.’

### Procedure

**FIGURE 1:**
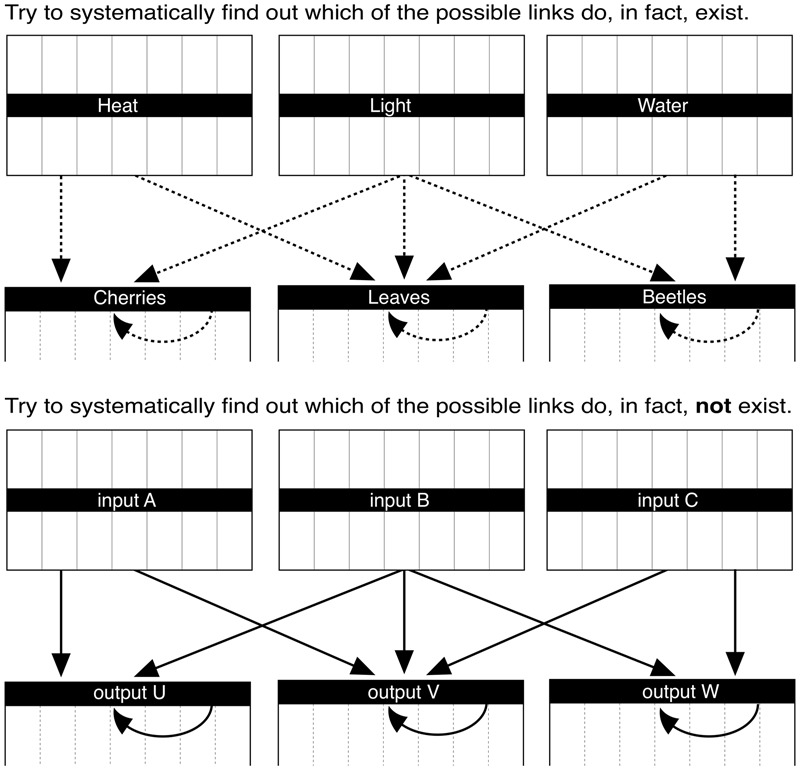
Partial screen captures of two of the four experimental conditions (**top**: compile condition for high semanticity; **bottom**: eliminate condition for low semanticity).

After completing a demographics questionnaire participants were randomly allocated to one of the four CPS conditions. The CPS systems were presented in a non-numerical, graphical format on the computer screen (see **Figure 1**). Prior to being instructed to start exploring the system under the assumption that either all or none of the relationships existed, participants allocated to the high semanticity condition (i.e., Cherry Tree) were asked to indicate their expectations regarding the causal structure that might underpin the system. This information was used to test whether the actual implemented causal structure could be perceived as counterfactual to “common” expectations.

*Phase 1 – Knowledge Acquisition:* Participants were first instructed to acquire knowledge of the system variables’ interconnectedness. To do so they were given two cycles with seven trials each where they could freely change the values of the three input variables in their respective system and observe the subsequent changes in the output variables. After each exploration trial participants were asked to record their insights regarding the causal structure of the system in form of a causal diagram presented on screen. After the first cycle of seven trials the values for the output variables were reset, the causal diagram, however, remained on the screen.

In the compile conditions, the initial causal diagram consisted of dotted arrows representing *possible* links (see left panel in **Figure 1** for the Cherry Tree version). Over the course of the knowledge acquisition phase these arrows had to be changed into either solid arrows (indicating assumed links) or deleted arrows (indicating assumed non-links).

In the eliminate conditions, the initial causal diagram comprised solid arrows for all *possible* relationships (see right panel in **Figure 1** for the Black Box version). During the process of knowledge acquisition in this condition, arrows linking variables that were in fact identified as being unrelated were expected to be deleted from the diagram leaving only those arrows in the causal diagram for which a link is assumed to exist.

**FIGURE 2:**
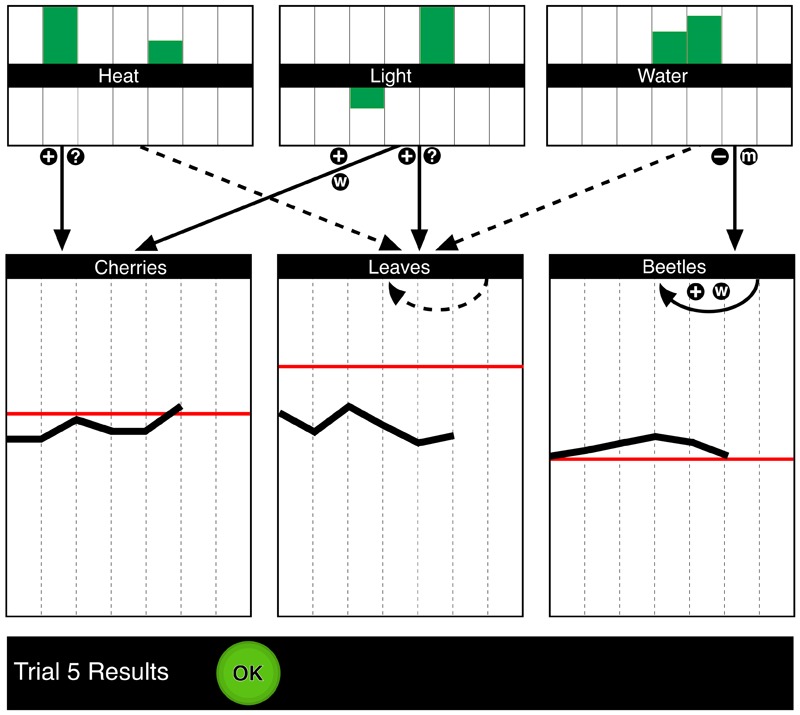
Screen capture from the control phase in the high semanticity condition.

*Phase 2 – Control*: In the second phase, participants were asked to control their respective system using their developed causal diagram, which represented their previously acquired causal knowledge. Participants had two control cycles with seven intervention trials each to reach and maintain two different target states, which were indicated as red horizontal lines in the respective panels of the output variables (**Figure 2**). After the first control cycle, the values for the output variables were reset and a different set of target values were given. Problem solvers were not informed about these target states prior to the respective control phase.

### Operationalizations

*Systematicity* of exploration behavior is operationalized via three ordinal categories. The intervention sequence necessary for being able to identify the underlying causal structure of the explored system comprises one exploration intervention where all inputs were left at zero followed by three exploration interventions where only one input was changed. Problem solvers who executed this sequence in this order at least once across their 14 exploration trials received a systematicity score of 2 (VONAT). Those who either failed to employ the zero intervention or where it did not precede the three single change interventions (i.e., traditional VOTAT) received a systematicity score of 1, otherwise a score of 0 was given. The rationale is that in systems with autoregressive dependencies an all preceding zero intervention is a necessary precondition to have the chance to correctly identifying direct effects using subsequent single change interventions.

*Knowledge acquisition*. The task of exploring a system to find out its underlying causal structure can be conceptualized as a “relationship detection task.” Taking CPS beyond a mere psychometric approach (i.e., by looking at more than the percentage of correctly identified relationships) should be reflected in the performance score used. We therefore based the operationalization of knowledge acquisition performance on a signal detection model that Snodgrass and Corwin (1988) introduced in the context of recognition memory. In this model the combined probability of correctly identifying existing and non-existing relationships (i.e., hits and correct rejections, resp.) form the sensitivity index *P*_r_ (Formula 1). Knowledge scores based on this operationalization have a theoretical range from -0.98 to 0.98, where a score below zero indicates inaccurate knowledge, whilst a score above zero indicates more accurate knowledge.

(1) $$P_{r}=(Hit rate)-(False Alarm\mathrm{rate})$$

In this model a Bias Index (*B*_r_) can also be derived, which reflects a problem solver’s tendency to either “see” or “not to see” relationships when in fact they are uncertain. Bias scores (*B*_r_, Formula 2) range from 0 to 1, where values below 0.5 indicate a conservative response tendency (i.e., “guessing that relationships do not exist”) and values above 0.5 indicate a liberal response tendency (i.e., “guessing that relationships exist”).

(2) $$B_{r}\;=\;\frac{False Alarm rate}{1-(Hit rate)-(False Alarm rate)}$$

*Control performance*. An operationalization of control performance by means of a simple metric of the distance between actual and target state after the final control intervention with limited reflection of the process, resembles the psychometric notion of a criterion-based assessment. That is, it does not differentiate between problem solvers who have reached the target state earlier and having to spend most of the time stabilizing the system, and problem solvers who reached the target closer to the end of the control cycle. As discussed in the context of measuring knowledge acquisition, given our aim to take CPS beyond a psychometric approach, the operationalization of control performance needs to better reflect how problem solvers cope with the cognitive demands (i.e., complexity) imposed by the start-target state discrepancy and the system characteristics (e.g., the dependency structure of output variables).

Finding the correct control intervention (i.e., set of inputs) that brings the system at or closest to the target state can be conceptualized as navigating the problem space. Different systems differ in their size and navigability as a function of (a) system characteristics such as the number and kind of dependencies, and/or (b) situational characteristics, such as the start-target discrepancy problem solvers must bridge. In order to allow for comparisons of performance scores across different studies, using different systems and/or different start-target discrepancies, performance scores need to be standardized against the size of the problem space of the respective system and start-target discrepancy. To achieve this standardization, we propose to operationalize control performance via the Euclidean distance between the intervention vector (i.e., values entered for the input variables) used by the participant and the vector of optimal interventions (i.e., inputs that would have brought the outputs at or closest to the target states^2^) for each trial of a control cycle (i.e., at each decision-input point). A standardization against the size of the problem space can be achieved by dividing the trial specific deviation scores by the trial specific difference between the vectors of pessimal and optimal intervention inputs [see formula (3)]. Consequently, control performance scores represent the averaged (across the seven control trials) deviation of the actual from the optimal intervention relative to maximal possible deviation for each and every trial. Their theoretical range is from 0 (worst possible, i.e., pessimal) to 1 (i.e., optimal).

(3) $$avEuXr=\frac{1}{m}\sum_{t=1}^{m}\left\{1-[\frac{\sqrt{\sum_{i=1}^{k}{\left({\mathrm{optimal}}_{\mathrm{ti}}-{\mathrm{actual}}_{\mathrm{ti}}\right)}^{2}}}{\sqrt{\sum_{i=1}^{k}{\left({\mathrm{pessimal}}_{\mathrm{ti}}-{\mathrm{optimal}}_{\mathrm{ti}}\right)}^{2}}}]\right\}$$

*m*: number of trials across control cycles (14 in this study),

*k*: number of input variables (three in this study).

## Results

The analyses are presented in two parts. First, we test two prerequisites, (1) the potential incompatibility of the underlying causal structure with the common expectation associated with a “real” cherry tree, and (2) the effectiveness of the instructions to start with the assumption that either all relationships existed or none of the relationships existed (manipulation check). In the second set of analyses we focus on the *Systematicity-Hypothesis* and the *Complexity–Difficulty Hypothesis*. **Table 1** provides an overview of the descriptive statistics in study-related variables across the experimental groups.

**Table 1:** Descriptive statistics.

Conditions			Variables			
Semanticity	Instruction	*N*	Bia *B*_r_[1] *M* (*SD*)	Systematicity [No,VOTAT,VONAT] frequencies	Knowledge acquisition *P*_r_[14] *M* (*SD*)	System control *avEuXr M* (*SD*)
Low (Black Box)	Compile	57	0.17 (0.19)	[3,28,26]	0.57 (0.30)	0.65 (0.12)
	Eliminate	59	0.89 (0.19)	[15,25,19]	0.41 (0.35)	0.60 (0.12)
High (Cherry Tree)	Compile	63	0.35 (0.27)	[18,33,12]	0.20 (0.25)	0.55 (0.09)
	Eliminate	61	0.87 (0.20)	[14,34,13]	0.33 (0.36)	0.59 (0.13)

As a first step, we tested whether a potential semanticity effect might simply be explained by the causal structure that underpins the CPS system being counterfactual to what one would expect in a “real” cherry tree. To this end we analyzed the problem solvers’ expectations regarding the causal structure of the Cherry Tree prior to being instructed to explore the system (i.e., using the Sensitivity Index *P*_r_ to operationalize prior expectations as prior knowledge). The resulting average *P*_r_(0) of -0.03 (*SD* = 0.21 based on *N*_CT_ = 124) indicates no systematic misalignment of common expectations with the actual causal structure (see **Figure A1**). In the case where the implemented system structure stood in contrast to common expectations (i.e., being “counterfactual”) the sensitivity index would have been substantially closer to -1.00. In cases where the implemented system structure would agree with a commonly held set of expectations – if such consensus existed in the first place – the resulting sensitivity index would be closer to +1.00. In the latter case, problem solvers would have already possessed knowledge that they were expected to acquire during the subsequent exploration phase. Both the average hboxtextitP_r_ value of around zero and the fact that expectations regarding the existence of relationships are equally distributed across the 12 possible variable links replicates what was found in earlier studies contrasting CPS scenarios with high and low semanticity (Beckmann, 1994; Beckmann and Goode, 2014, 2017). A counterfactual causal structure can therefore be ruled out as an alternative explanation for a potential semantic effect.

**FIGURE 3:**
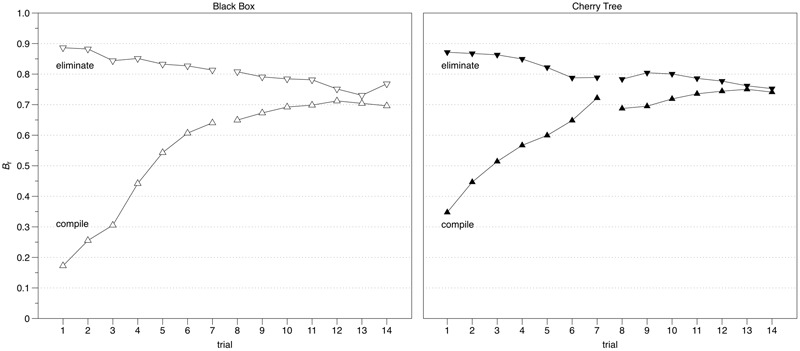
Comparison of bias (*B*_r_) trajectories between the two instruction conditions for each of the two semanticity conditions.

Instruction Manipulation: In a next step, we checked whether the instruction to start with the assumption that either all relationships existed (eliminate condition) or none of the relationships existed (compile condition) was reflected in problem solvers’ response behavior during the knowledge acquisition phase. Problem solvers’ ability to follow these instructions should be identifiable in the trajectories of the bias scores (*B*_r_) over the course of the two exploration cycles with their seven trials each. We expect problem solvers in the eliminate condition to start with a bias score greater than 0.5 and close to 1.00 as this would indicate an instruction-induced tendency “*to guess that there is*” a relationship when in fact (still) in a state of not knowing. Problem solvers in the compile condition, however, were expected to start with a conservative bias (i.e., a *B*_r_ score below 0.5 and close to zero), which would indicate a response tendency of “*guessing that there is not*” a relationship when in the state of (yet) not knowing. In both conditions, we expected bias scores to become more neutral (i.e., *B*_r_ ≈ 0.5) over the course of the exploration trials and when progressing in acquiring knowledge. The left panel in **Figure 3** depicts the differing bias trajectories for the “Black Box” conditions; the right panel shows them for the “Cherry Tree” conditions. It is interesting to note that the final convergence occurs at a level of around 0.75 for all conditions. This seems to indicate a general propensity to slightly err on the positive, i.e., to rather assume that there are relationships than running the risk of missing one.

The trajectories seem to suggest that the instruction has led to the expected differences in response behavior, confirming the effectiveness of the instruction manipulation, in general. Two further suggestions seem to emerge. First, the slopes for the eliminate conditions are markedly less steep than the ones for the compile conditions (*F*_4.1,968.5_ = 76.455, *p* < 0.001, η^2^ = 0.224)^3^, which seems to suggest that reducing complexity is more challenging than increasing it, regardless of semanticity. Second, the starting point of the compile condition for “Cherry Tree” is not as low as it is for “Black Box” (*B*_r_[1]: *t*_118_ = 4.13, *p* < 0.001, *d*_compile-BBvsCT_ = 0.76), which seems to suggest that adopting a “blank slate” perspective is more challenging in a system with high semanticity.

### Systematicity

To test the Systematicity-Hypothesis we conducted an ordinal logistic regression analysis where problem solvers’ VONAT score was regressed on the semanticity condition and the instruction condition they have been allocated to. The results indicate (see **Table 2**) that problem solvers who were asked to explore a system with low semanticity (i.e., Black Box) were 2.24 time more likely to employ a systematic exploration heuristic (i.e., using VOTAT or VONAT) than problem solvers working on a system that used variables labels high in semanticity (i.e., Cherry Tree). Being instructed to either systematically eliminate erroneously presumed relationships or to identify existing relationships in the causal model of the respective system did not, however, make a substantial difference in the level of systematicity with which problem solvers explored the system.

**Table 2:** Ordinal logistic regression of systematicity (VONAT) on semanticity and instruction.

	Estimate	*SE*	Wald χ^2^	*df*	*p*	Odds ratio
Semanticity (Black Box vs. Cherry Tree)	0.804	0.251	10.256	1	0.001	2.24
Instruction (compile vs. eliminate)	-0.307	0.246	1.557	1	0.212	0.74

### Complexity – Difficulty

**FIGURE 4:**
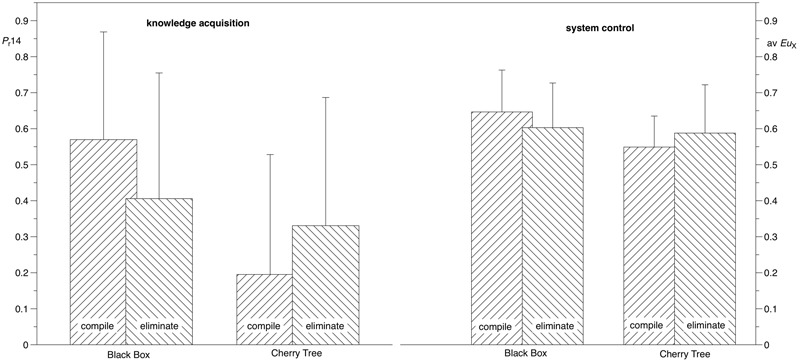
Disordinal interaction effects of semanticity and instruction on knowledge acquisition **(left)** and system control **(right)**.

To address the *Complexity–Difficulty Hypothesis* we tested in a final step whether the effect of the instruction differs between the two levels of semanticity in terms of the knowledge acquisition performance and control performance metrics. Given both metrics produced comparable effects and interpretations, we report them together. As expected, the ANOVAs resulted in a main effect of the situational factor “semanticity,” with overall lower performance scores (knowledge acquisition: *F*_1,236_ = 29.863, *p* < 0.001, η^2^ = 0.11; control performance: *F*_1,236_ = 14.048, *p* < 0.001, η^2^ = 0.06) for the high semanticity condition (i.e., Cherry Tree) relative to the low semanticity condition (“Black Box”). This replicates the semantic effect once more (Beckmann, 1994; Beckmann and Guthke, 1995; Beckmann and Goode, 2014). Across the two semanticity conditions, the task factor “instruction” seems to have no effect on performance scores overall (knowledge acquisition: *F*_1,236_ = 0.119, *p* = 0.730, η^2^ ≈ 0.00, control performance: *F*_1,236_ = 0.027, *p* = 0.870, η^2^ ≈ 0.00). However, the presence of an interaction effect (knowledge acquisition: *F*_1,236_ = 13.235, *p* < 0.001, η^2^ = 0.05, control performance: *F*_1,236_ = 7.544, *p* = 0.006, η^2^ = 0.03), indicates that when being instructed to start with the assumption that all relationships existed and consequently systematically eliminate unjustified presumptions showed a positive effect on both knowledge acquisition and control performance in conditions of high semanticity (i.e., Cherry Tree), but it resulted in systematically lower performance scores in the condition where problem solvers were working with low levels of semanticity (i.e., Black Box, see **Figure 4**).

### Summary

Knowledge acquisition, especially in systems with high levels of semanticity, can be conceptualized as a process of transforming a presumption structure into a knowledge structure. The instruction to start with the assumption that all possible relationships between system variables existed aimed at creating a presumption structure with high levels of complexity. If we were to use the number of relationships in a system (*NoR*) as a crude quantifier of complexity (see Beckmann and Goode, 2017 for a more detailed discussion) the process of knowledge acquisition under this instruction requires the reduction of complexity from a *NoR*_presumed_ = 12 to *NoR*_actual_ = 6. In contrast, the instruction to start with a “blank slate” (i.e., assuming that no relationship exists) aimed at creating a situation where complexity needed to be increased from *NoR*_presumed_ = 0 to *NoR*_actual_ = 6. The slope differences in bias scores between the two instruction conditions suggest that decreasing the complexity of a presumption structure is more challenging than is building up the complexity of a knowledge structure, regardless of the semanticity of the explored system.

If we were to interpret the difference in the initial Bias-scores between the two instruction conditions as an indicator of how well problem solvers were able to adopt a “full slate” or “blank slate” perspective then the significant interaction effect between semanticity and instruction would indicate that the *instruction–adoption* differs between the two semanticity conditions. Problem solvers tend to struggle adopting a “blank slate” perspective under the high semanticity condition. From a cognitive task analysis point of view, we could surmise that adopting a “blank slate” perspective under high semanticity conditions requires the suppression of preconceived expectations regarding the causal structure of the system as they are triggered by the semanticity of the variable labels. The process of suppression or decontextualization seems to add to the *complexity* of the task of knowledge acquisition in CPS-systems high in semanticity. In short, *semanticity*, as a situational characteristic of CPS, has the potential of being a source of *complexity*.

We have also argued that systematicity (i.e., the creation of informative mini-experiments that help to identify the existence or non-existence of relationships between system variables) is a necessary precondition for successful knowledge acquisition, independent of instruction conditions or semantic embedment of the system. Our findings, however, suggest that problem solvers working under high semanticity conditions are on average less likely to engage in systematic exploration behavior. At this stage, it is difficult to conceive of a “cognitive argument” that would predict that the heuristic of systematically *testing against* presumptions (as required in the eliminate conditions) is cognitively more demanding than *testing for* evidence of the existence of relationships (as would be required in the compile conditions). This, in conjunction with the fact that problem solvers in the compile condition with low semanticity were able to be more systematic, leads to the conjecture of seeing the failing to employ a suitable or necessary heuristic as an indication of the greater *difficulties* problem solvers seem to have. In short, semanticity as a situational characteristic of CPS might also be a potential source of (unnecessary) *difficulty*.

In switching the focus from the bias score (indicating the adoption of the instructed behavior) and systematicity score (indicating the level of engaging in planned and coordinated behavior) onto performance (i.e., knowledge acquisition as well as control), the data suggest that in conditions of high semanticity, it is more effective to start with the presumption that all relationships might exist (“full slate”) rather than to start pretending that none exist. This requires systematic *testing against* a priori assumptions regarding the system’s underlying causal structure, with the emphasis on “systematic.” In conditions with low semanticity, however, it seems *less* effective to start with presumptions of existing relationships (it is safe to assume that such presumptions would likely be a result of conscious efforts to guess). The more “natural” starting position here would be something more akin to a “blank slate” (or, knowing that one does not know), which then would require a systematic *testing for* evidence regarding the system’s underlying causal structure.

The fact that knowledge acquisition and control performances in the high semanticity conditions still fall short of those shown in low semanticity conditions (i.e., replicating the “semantic effect”) can be explained via two factors. First a *complexity factor*, which reflects the additional cognitive demands associated with suppressing presumptions when trying to adopt a “blank slate” starting position under high semanticity conditions, and second a *difficulty factor*, which reflects the tendency of problem solvers to not adopt a systematic approach to exploration behavior.

## General Discussion

These reflections should not be misunderstood as an unconditional plea against the use of semantically laden variable labels in CPS. The answer to the question of what kind of systems should be used is once more the infamous: it depends. It depends on the purpose of the use of CPS scenarios. If, for instance, we aim to measure problem solvers’ ability to draw inferences based on observed outcomes of systematic experimentation, we need to consider that arguably minor changes in situational characteristics, such as the semanticity of variable labels, have the potential to prevent the spontaneous employment of systematic experimentation (see also Beckmann and Goode, 2014). Under these circumstances, it would be inappropriate to interpret performance scores as indicators of problem solvers’ reasoning ability or to expect them to correlate highly with reasoning measures. If, however, the aim was to predict “real life decision making” and given that “real life problems” are always semantically anchored, then using systems with high semanticity might be appropriate. The “construct purity” (or uni-dimensionality, in psychometric terms) of the measure, however, is likely to be compromised, which needs to be reflected (a) in expectations regarding inter-test correlations and (b) in the way performance scores are interpreted. Schoppek and Fischer (2015) make a convincing case for conceptualizing CPS performance scores as indicators of a competency, whereby a competency is a conglomerate of knowledge, reasoning ability, thinking skills and motivational variables. The PTS framework proposed here can help draw attention to the often-overlooked potential impact that situational characteristics might have on the composition of knowledge, reasoning ability, thinking skills and motivational variables in performance scores obtained from dealing with supposedly homomorphous CPS systems. For instance, the practice of aggregating performance scores obtained in multiple minimal complex systems (e.g., Funke, 2014; Stadler et al., 2016) with various levels of semanticity might be psychometrically desirable (e.g., maximizing reliability). At the same time, however, this very practice could (inadvertently) turn out to be a threat to construct validity if performance scores are underpinned by qualitatively different cognitive processes (e.g., compiling vs. eliminating), varying levels of functional or dysfunctional prior knowledge, and/or differences in perceived personal relevance of the semantic context these systems are embedded in. The PTS framework might also be (in-)formative for on-going discussions as to whether CPS performance scores are more than *g* or not (e.g., Gonzales et al., 2005; Wüstenberg et al., 2012; Hundertmark et al., 2015; Schoppek and Fischer, 2015; Stadler et al., 2015).

The use of semantically laden cover stories or variable labels to induce a stronger sense of “real life” relevance of the CPS experience for the participants in our laboratory studies or large scale assessment exercises should also not be mistaken as a shortcut to what some might call ecological validity. If we were to define ecological validity as the meaningfulness, appropriateness and usefulness of inferences drawn based on performance scores obtained using an assessment tool, one would have to convincingly demonstrate that the problems posed in the assessment situation have triggered the same cognitive or affective processes as they are expected to be involved when dealing with complexity, uncertainty and dynamics in the “real world.” Otherwise we run the risk of simply falling prey to our own make-believe. Proper validation requires an *ex ante* specification of the cognitive or affective processes expected to be involved when dealing with complexity, uncertainty and dynamics in the “real world.” The distinction between complexity and difficulty, as being proposed here, can help moving beyond psychometrics-driven *post hoc* interpretations of mean scores and correlation patterns.

The differentiation between complexity and difficulty can also help improving the conceptual and psychometric quality of the assessment or research tools we use in the context of CPS. For instance, result patterns indicating that problem solvers overall experienced fewer difficulties (i.e., better performance) than the ex ante specifications of complexity would have led us to expect, could suggest that problem solvers might not have had to engage in the sequences of cognitive processes anticipated. Certain task-independent situational features could have enabled the use of prior knowledge or chunked information cues (Wood, 1986) and consequently created “construct-irrelevant easiness” (Messick, 1995). Conversely, “construct-irrelevant difficulty” (Messick, 1995) could result from a misalignment between (empirically observed) performance scores and (theoretically pre-determined) complexity specifications, where the former is systematically lower than the latter would have suggested. This could have been triggered by situational variables (inadvertently) preventing problem solvers from engaging in the anticipated sequence of cognitive behaviors. Both instances present a threat to validity that might be overlooked if complexity and difficulty are treated synonymously.

The main intent of this paper was to contribute to the discussion around taking CPS beyond a narrowly defined psychometric approach. We are of the view that a predominantly psychometric perspective tends to fall short in appropriately capturing the essence of CPS, namely complexity. We identified the lack of a differentiation between complexity and difficulty as a major barrier to achieving conceptual progress in CPS research. To redress this, we introduced the Person–Task–Situation (PTS) framework which, through the theoretical distinction it makes between its constituent factors, enables a conceptual differentiation of complexity and difficulty. The differentiation provides a theory-based platform for studying cognition (e.g., information processing, learning, decision making, reasoning) beyond an atheoretical psychometric lens.

Complexity as a concept also includes a qualitative dimension, whilst difficulty is exclusively quantitative. Complexity is a cognitive concept that reflects the interactive effects of information processing demands imposed upon the cognitive system by task and situation characteristics (i.e., the T and the S in the PTS framework). Difficulty is a psychometric concept that reflects the level of success problem solvers have in dealing with complexity. The integration of the person (i.e., the P in the PTS framework) introduces individual differences in ability, memory, knowledge and attitudinal variables as potential explanatory factors for observed performance differences. Cognition research in general, and CPS research in particular, focuses on studying the links between complexity and difficulty. By ignoring their conceptual differences and treating them synonymously, CPS research runs the risk of loosing sight of its cognition-based origins and failing to utilize its potential.

As a case in point, we used the “semantic effect” to test these conceptualizations. We were able to show that by using the same system (i.e., keeping the *task qua task* constant) and asking problem solvers to freely explore the system to find out its underlying causal structure (i.e., keeping the *task as behavior requirement* constant), but varying the system’s semantic embedment via using different variable labels (i.e., varying a *situational* variable) systematic differences in exploration behavior occurred. Failing to differentiate task and situation as independent sources of complexity and by treating complexity and difficulty synonymously the resulting performance differences would erroneously be attributed to individual differences in person-related variables.

The conceptual distinction between complexity and difficulty paves the path for taking CPS beyond a psychometric approach. In fact, it is instrumental to bringing the “psycho-” back into psychometric. Otherwise one tends to operate with a “metric” that is agnostic to theory, and can therefore not be scrutinized for validity. The validity question is the core element of empirical research in psychology that relies on a strong conceptual underpinning. Psychometrics is a tool for linking the theoretical and the empirical and should not be used as a substitute for either.

The study presented here is not intended as a comprehensive test of the PTS framework that underpins the complexity–difficulty distinction. Instead, the paper should be considered as an invitation and orientation for future work. The theoretical analyses and empirical outcomes we report support the proposed complexity framework in demonstrating that it is both specific enough to allow for testable hypotheses, yet broad enough to allow modifications and refinements. Our work also contributes to efforts to better understand the person–task–situation tripartite. Future conceptual and empirical contributions will be necessary to further develop and refine a common framework that considers the interplay of the person, the task and the situation and has complexity at its conceptual core. This, so we have argued, is particularly pertinent to a research paradigm such as CPS that carries complexity in its label. Efforts to this end will support the better integration of research findings from existing and future studies on CPS.

## Ethics Statement

This study has been reviewed by, and received ethics clearance through, the Human Research Ethics Committee (HREC), University of New South Wales, Australia (Approval No: 09616 and 06294). After being informed (a) what participation in the study entails, (b) that participation was voluntary and (c) withdrawal from participation was possible at any time without negative consequences, and (d) that anonymity was guaranteed, participants were asked to sign an informed consent form prior to participation.

## Author Contributions

JB, DB, and NG certify that they have participated sufficiently in the work to take responsibility for the content, including participation in the conception, design, analysis, drafting the work, writing, and final approval of the manuscript. Each author agrees to be accountable for all aspects of the work.

## Conflict of Interest Statement

The authors declare that the research was conducted in the absence of any commercial or financial relationships that could be construed as a potential conflict of interest.

## Footnotes

1 We emphasize *expected* here because it highlights that complexity is based on *ex ante* developed expectations regarding the set of cognitive behaviors necessary to deal with the challenge posed by the task in conjunction with the situation.

2 The vector of ideal inputs would bring the system exactly to (or would maintain) the target state. Restrictions of the range of possible input variables (e.g., introduced for practical reasons) might prevent reaching the target state in any one single intervention. In such cases (i.e., the ideal values fall outside this range), the values were adjusted to the nearest possible values, and these then constituted the vector of optimal inputs. In cases when the ideal values are within the range of possible inputs, the ideal values were used as the optimal input.

3 Greenhouse-Geisser correction of *df*s for the *F*-test was applied due to sphericity (χ^2^_90_ = 2201.984, *p* < 0.001).
